# Proposal for a Histological Staging System of Mammary Carcinomas in Dogs and Cats. Part 1: Canine Mammary Carcinomas

**DOI:** 10.3389/fvets.2019.00388

**Published:** 2019-11-07

**Authors:** Florian Chocteau, Jérôme Abadie, Delphine Loussouarn, Frédérique Nguyen

**Affiliations:** ^1^AMaROC (Animal Cancers, Models for Research in Comparative Oncology), Oniris, Nantes Atlantic College of Veterinary Medicine, Food Science and Engineering, Nantes, France; ^2^CRCINA, INSERM, Université d'Angers, Université de Nantes, Nantes, France; ^3^Department of Pathology, University Hospital, Nantes, France; ^4^Integrated Center for Oncology Nantes/Angers, Nantes, France

**Keywords:** dog, lymphovascular invasion, mammary carcinoma, pathologic nodal stage, pathologic tumor size, prognosis, stage, survival

## Abstract

**Background:** Staging of mammary carcinomas of dogs and cats is not only important for prognostic purposes, but also to guide therapy, in particular regarding adjuvant chemotherapy. The classical staging system relies on T, the clinical tumor size, N, the clinical nodal stage, and M, distant metastasis, evaluated by the clinician. However, a more precise and reliable staging system is applied to human stage I–III breast cancer, i.e., without distant metastasis, in which T is replaced by the pathologic tumor size (pT), and N is replaced by the pathologic nodal stage (pN), both evaluated by the pathologist. This staging system is strongly associated with patient outcomes, and is used to select treatment options. The purpose of this study was to design a histologic staging system for Canine Mammary Carcinomas (CMCs, part 1 of this article), and Feline Mammary Carcinomas (part 2), inspired from human oncology, and to assess its association with patient outcomes.

**Materials and Methods:** This retrospective study included 433 female dogs with a surgically removed CMC. Patient outcomes were recorded over a 2-years follow up period. CMCs were staged according to pT (greatest diameter in millimeters on histological slides), lymphovascular invasion (LVI), and pN (confirmed by cytokeratin AE1/AE3 immunohistochemistry). The histological stages were defined as: Stage 0 (CMCs *in situ*, surrounded by a continuous layer of p63+ myoepithelial cells), Stage I (pT1 ≤ 20 mm, LVI–, pN0–pNX, where pNX refers to the absence of lymph node sample), Stage II (pT2 > 20 mm, LVI–, pN0–pNX), Stage IIIA (pT1, LVI+, and/or pN+), and Stage IIIB (pT2, LVI+, and/or pN+).

**Results:** Disease-free-interval, overall survival and specific survival significantly differed by histological stage. For specific survival, median survival times and hazard ratios (HR) by Cox proportional hazards regression (*p* < 0.0001) were: Stage 0 (median survival not reached; HR = 1.00; *N* = 89; 21% of the dogs), Stage I (1,720 days; HR = 3.05; *p* = 0.0018; *N* = 81; 19%), Stage II (1,181 days; HR = 4.39; *p* < 0.0001; *N* = 79; 18%), Stage IIIA (348 days; HR = 10.59; *p* < 0.0001; *N* = 79; 18%), and Stage IIIB (163 days; HR = 16.59; *p* < 0.0001; *N* = 105; 24%).

**Conclusion:** The proposed histological staging system (invasiveness, pT, LVI, pN) is a very strong prognostic factor for CMCs.

## Introduction

Canine Mammary Carcinomas (CMCs) are the most common tumors in female dogs, with an estimated annual incidence of 192 cases per 100,000 bitches (1–3). One of the difficulties in assessing prognosis results from CMCs being characterized by very different initial presentations and biological behaviors, ranging from small non or weakly invasive tumors to large invasive cancers with nodal and/or distant metastases (4, 5).

In order to define the degree of local, regional, and distant tumor extent within canine patients, a staging system has been historically established based on the clinical parameters “TNM” (6): the clinical tumor size (T) with thresholds of 3 and 5 centimeters, the presence of nodal metastasis (N), and distant metastasis (M) diagnosed by palpation, medical imaging, biopsy or cytology. Four (7) or five (6) stages are thus recognized for CMCs, which have been significantly associated with prognosis by survival analyses (5, 8–11). The clinical staging system for CMCs is also used in order to adapt treatment (6, 11, 12). Among therapeutic possibilities, surgical resection is always the first-line choice, and allows defining the degree of malignancy and histological type of the tumor. Adjuvant chemotherapy is mostly indicated for CMCs with positive lymph node involvement (6).

New developments of the TNM staging system for CMCs now appear in veterinary medicine, which use histopathology and/or immunohistochemistry in addition to clinical parameters. First, immunohistochemical methods using myoepithelial cell markers enable the distinction between mammary carcinomas *in situ*, i.e., carcinomas restricted to the pre-existing limits of mammary lobules and ducts, from invasive mammary carcinomas, i.e., infiltrating carcinomas with the possibility of metastatic spread (13–16). This distinction is of paramount importance in human oncology, as treatment modalities and follow-up guidelines substantially differ between patients with mammary carcinomas *in situ* and those with invasive breast cancer (17). Thus, stage 0 breast cancer (mammary carcinoma *in situ*) is recognized as a separate stage in the breast cancer staging system published by the American Joint Committee of Cancer (18).

Secondly, it is possible to replace the clinically measured tumor size (T) by the pathologic tumor size (pT), i.e., the largest diameter of the CMC (in millimeters) measured on histological slides or on formalin-fixed resected samples (19). Compared to T, pT does not take into account skin thickness and eventual mammary hyperplasia adjacent to the carcinoma; thus, pT is smaller, but more precise, than T.

Thirdly, the clinical nodal status (N) may be replaced by the pathologic nodal stage (pN), relying on histologic examination of the regional lymph node, and immunohistochemistry to epithelial markers if necessary. The draining lymph node of a CMC may contain macrometastases (>2 mm in diameter), micrometastases (0.2–2 mm in diameter), or isolated tumor cells (<0.2 mm in diameter or <200 cells) (20, 21), as defined in breast cancer (18). In human oncology, this pathologic nodal stage is first defined on the sentinel lymph node, i.e., the first lymph node to which the mammary carcinoma drains, but sentinel lymph node mapping is not yet routinely performed in veterinary oncology (22).

Moreover, there are still substantial numbers of CMC cases in which the draining lymph node was not sampled during mastectomy, thus compromising nodal staging (19). In these instances, it is tempting to use lymphovascular invasion (LVI), the presence of tumor emboli within lymph and/or blood vessels, as a surrogate for nodal stage, as LVI has been identified as a predictor of lymph node status in bitches with CMC (23), and is a strong prognostic factor of CMCs (5, 11, 19, 23, 24).

The objectives of this study were (1) to design a histological staging system for female dogs with CMC, which would be easily assessed by veterinary pathologists, and (2) to validate its prognostic value in terms of patient survival. Inspired from human breast cancer staging, the proposed histological staging system for CMCs identifies a stage 0 category corresponding to mammary carcinomas *in situ*, which may not represent indications for adjuvant chemotherapy, and uses LVI as a complement of lymph node evaluation in order to define the regional spread of canine invasive mammary carcinomas.

## Materials and Methods

### Patients and Follow-Up

This retrospective study included 433 female dogs diagnosed with mammary carcinoma, of which 89 had a mammary carcinoma *in situ*, and 344 had an invasive mammary carcinoma, and were previously described (19, 25). The owners' written consent and approval from the Oniris College of Veterinary Medicine local Animal Welfare Committee were obtained prior to inclusion. Female dogs were eligible for inclusion when they had a surgically removed mammary carcinoma and at least a 2-years follow-up after diagnosis. Incomplete records, the presence of distant metastasis at initial presentation, the presence of another malignant tumor, or administration of adjuvant treatments prior or after surgery were exclusion criteria. Age, breed, reproductive and medical history, and outcome were obtained through written questionnaires or telephone interviews with referring veterinarians and owners. The outcome data included disease-free interval (DFI, interval from mastectomy to the first local recurrence, new primary tumor, lymph node metastasis, and/or distant metastasis), overall survival (OS, time from mastectomy to death from any cause), and cancer-specific survival (SS, time from mastectomy to death attributable to the mammary carcinoma).

### Conventional Histopathology

Histological examination was performed on 3-μm-thick hematoxylin–eosin-saffron (HES) stained whole sections (not partial biopsies) of mammary carcinomas. Recorded data included histological types according to the adapted World Health Organization classification system (26, 27), histological grades according to modified Elston and Ellis' criteria (9, 27), lymphovascular invasion (LVI), local invasion of dermis or muscle, margin status, tumor-associated inflammation, central necrosis, ulceration, and squamous differentiation, as previously described (19). The pathologic tumor size (pT) was measured on HES-stained sections as the greatest tumor diameter, in millimeters. In our laboratory for histopathology, tumors that measure <25 mm after formalin fixation (i.e., approximate inside dimensions of a tissue embedding cassette) are bisected along their longest axis, allowing for visualization of their largest dimension on histological slides. For tumors that measure 25–50 mm after formalin fixation, sectioning is also performed along their longest axis, and then two halves of the tumor are placed in two separate cassettes; tumor size was thus the sum of tumor length measured on the slides obtained from these two paraffin blocks. For tumors larger than 50 mm in diameter after formalin fixation, the pathologic tumor size could not be precisely determined.

### Immunohistochemistry

Immunohistochemistry (IHC) was performed using a Benchmark XT automated instrument (Ventana Medical Systems, Roche Diagnostics) as previously described (19, 25). IHC to the myoepithelial marker p63 (mouse monoclonal, clone 4A4, abcam, dilution 1:100) was used to differentiate mammary carcinomas *in situ* (surrounded by a continuous layer of p63+ myoepithelial cells) from invasive mammary carcinomas (lacking a continuous layer of p63+ myoepithelial cells), as performed in human breast cancer (28), and validated in canine mammary carcinomas (13). In the absence of any metastatic carcinoma cells in the draining lymph node on HES-stained sections, IHC to pancytokeratin (mouse monoclonal, clones AE1/AE3, Dako, dilution 1:200) was performed on the draining lymph node (29), to identify potential isolated tumor cells or micrometastases. A lymph node was considered as metastatic (pN+, positive nodal stage) if there was *a minima one* epithelial tumor cell within (isolated tumor cells, micrometastases and macrometastases).

Immunophenotypes were determined using antibodies to Estrogen Receptor alpha (ER, mouse monoclonal, clone C311, Santa Cruz, dilution 1:50), Progesterone Receptor (PR, rabbit monoclonal, clone 1E2, Roche Diagnostics, prediluted), Human Epidermal growth factor Receptor Type 2 (HER2, rabbit monoclonal, clone 4B5, Roche Diagnostics, prediluted), and Ki-67 (mouse monoclonal, clone MIB1, Dako, dilution 1:50), as previously described (19). For invasive mammary carcinomas, IHC to cytokeratins 5 and 6 (CK5/6, mouse monoclonal, clone D5/16B4, Dako, dilution 1:50), and Epidermal Growth Factor Receptor Type 1 (EGFR, mouse monoclonal, clone 31G7, Invitrogen, dilution 1:20) were also performed.

Thresholds for positivity were ≥10% for ER and PR (14, 25, 30), CK5/6, and EGFR (31), and ≥20% for the proliferation index Ki-67 (32, 33). HER2 was scored according to the American Society of Clinical Oncology guidelines for breast cancer (34). Carcinomas were considered HER2 positive only for a 3+ IHC score (14).

CMCs were then defined as luminal (ER ≥ 10% and/or PR ≥ 10%, HER2 score 0 to 2+) or triple-negative (ER <10%, PR <10%, HER2 scores 0–2+).

Four veterinary pathologists and one medical pathologist examined the HES and IHC slides blindly. In case of discrepancy, cases were collectively reviewed in order to achieve a consensual diagnosis, grade, and immunohistochemical scoring.

### Histological Staging System

The histological stages (Table 1) were defined as: Stage 0 (mammary carcinomas *in situ*, surrounded by a continuous layer of p63+ myoepithelial cells by immunohistochemistry), Stage I [invasive, pathologic tumor size ≤ 20 mm (pT1) with a negative or unknown nodal status (pN0–pNX) and without lymphovascular invasion, LVI–], Stage II [invasive, pathologic tumor size >20 mm (pT2), pN0–pNX nodal status, and LVI–], Stage IIIA [invasive, pT1, with a positive nodal stage (pN+) and/or presence of lymphovascular invasion], and Stage IIIB [invasive, pT > 20 mm (pT2), LVI+, and/or pN+].

**Table 1:** Histological staging system proposed for canine mammary carcinomas.

**Stage**	**Invasiveness**	**Pathologic tumor size**	**Lymphovascular invasion**	**Pathologic nodal stage**
Stage 0	*In situ*	Any pT	LVI–	pN0 or pNX
Stage I	Invasive	pT1 ≤ 20 mm	LVI–	pN0 or pNX
Stage II		pT2 > 20 mm		
Stage IIIA		pT1 ≤ 20 mm	LVI+ and/or pN+	
Stage IIIB		pT2 > 20 mm		

*LVI–, absence of lymphovascular invasion*.

*LVI+, presence of lymphovascular invasion*.

*pN0, absence of nodal metastasis*.

*pN+, presence of nodal metastasis*.

*pNX, nodal stage unknown*.

### Statistical Analyses

The MedCalc® statistical software (Ostend, Belgium) was used. Continuous variables are expressed as median, range, mean ± standard deviation. Correlations between categorical variables were analyzed using the Pearson Chi^2^ test. The Kaplan–Meier method and log-rank tests were used for univariate survival analyses, and Cox proportional hazards models for multivariate survival analyses, whose results are reported using the Hazard Ratio (HR), its confidence interval (95% CI), and the *p*-value of each covariate. For all statistical tests, a *p*-value <0.05 was considered significant.

## Results

### Patients Characteristics

Four hundred and thirty-three bitches fulfilled the inclusion criteria, and their detailed characteristics are shown in Table 2.

**Table 2:** Initial presentation by stage.

**Parameter**		**Total *N* = 433**	**Stage 0 *N* = 89**	**Stage I *N* = 81**	**Stage II *N* = 79**	**Stage IIIA *N* = 79**	**Stage IIIB *N* = 105**	***p*-Value**
Age (years)	Mean ± SD	10.7 ± 0.1	9.7 ± 0.2	10.9 ± 0.2	10.5 ± 0.2	11.3 ± 0.2	11.1 ± 0.2	<0.0001^a^
Hormonal status	Intact female	324 (74.8%)	76 (85.4%)	56 (69.1%)	60 (75.9%)	53 (67.1%)	79 (75.5%)	NS 0.055^b^
	Neutered female	109 (25.2%)	13 (14.6%)	25 (30.9%)	19 (24.1%)	26 (32.9%)	26 (24.5%)	
Contraception	No or unknown	405 (93.6%)	81 (91.0%)	71 (87.7%)	76 (96.2%)	76 (96.2%)	101 (96.1%)	NS 0.070^c^
	Yes	28 (6.4%)	8 (9.0%)	10 (12.3%)	3 (3.8%)	3 (3.8%)	4 (3.9%)	
Multicentricity	Yes	59 (13.6%)	7 (7.9%)	14 (17.3%)	10 (12.7%)	14 (17.7%)	14 (13.3%)	NS 0.103^b^
	No	374 (86.4%)	82 (92.1%)	67 (82.7%)	69 (87.3%)	65 (82.3%)	91 (86.7%)	
Location^d^	M1–M2	68 (17.5%)	18 (23.4%)	17 (23.9%)	12 (16.0%)	11 (15.3%)	10 (10.8%)	NS 0.181^b^
	M3–M5	309 (79.6%)	59 (76.6%)	51 (71.8%)	62 (82.7%)	58 (80.6%)	79 (84.9%)	
	Thoraco-abdominal	11 (2.9%)	0 (0%)	3 (4.2%)	1 (1.3%)	3 (4.1%)	4 (4.3%)	
Inflammation	Moderate to severe	185 (42.7%)	19 (21.3%)	24 (29.6%)	33 (41.8%)	46 (58.2%)	63 (60.0%)	<0.0001^b^
	Absent to mild	248 (57.3%)	70 (78.7%)	57 (70.4%)	46 (58.2%)	33 (41.8%)	42 (40.0%)	
Central necrosis	Yes	326 (75.3%)	70 (78.7%)	56 (69.1%)	68 (86.1%)	54 (68.4%)	78 (74.3%)	NS 0.056^b^
	No	107 (24.7%)	19 (21.3%)	25 (30.9%)	11 (13.9%)	25 (31.6%)	27 (25.7%)	
Margins	Negative	251 (58.0%)	62 (69.7%)	69 (85.2%)	47 (59.5%)	39 (49.4%)	34 (32.4%)	<0.0001^b^
	Positive	182 (42.0%)	27 (31.3%)	12 (14.8%)	32 (40.5%)	40 (50.6%)	71 (67.6%)	
Histological grade	I	78 (18.0%)	59 (66.3%)	16 (19.8%)	1 (1.3%)	1 (1.3%)	1 (1.0%)	<0.0001^b^
	II	130 (30.0%)	26 (29.2%)	28 (34.6%)	24 (30.4%)	29 (36.7%)	23 (21.9%)	
	III	225 (52.0%)	4 (4.5%)	37 (45.7%)	54 (68.3%)	49 (62.0%)	81 (77.1%)	
ER	Mean index (%) ± SD	7.3 ± 13.1	11.7 ± 10.8	6.2 ± 11.8	5.6 ± 13.2	7.7 ± 15.0	5.3 ± 13.6	0.005^a^
	ER– (<10%)	338 (78.1%)	50 (56.2%)	64 (79.0%)	69 (87.3%)	62 (78.5%)	93 (88.6%)	<0.0001^b^
	ER+ (≥10%)	95 (21.9%)	39 (43.8%)	17 (21.0%)	10 (12.7%)	17 (21.5%)	12 (11.4%)	
PR	Mean index (%) ± SD	7.2 ± 14.9	14.0 ± 12.7	10.0 ± 21.2	5.2 ± 13.0	4.2 ± 13.2	3.4 ± 10.3	<0.0001^a^
	PR– (<10%)	348 (80.4%)	44 (49.4%)	65 (80.2%)	71 (89.9%)	71 (89.9%)	97 (92.4%)	<0.0001^b^
	PR+ (≥10%)	85 (19.6%)	45 (50.6%)	16 (19.8%)	8 (10.1%)	8 (10.1%)	8 (7.6%)	
HER2	Score 0	293 (67.7%)	50 (56.2%)	62 (76.5%)	54 (68.4%)	54 (68.3%)	73 (69.5%)	NS 0.089^b^
	Score 1+	108 (24.9%)	33 (37.1%)	15 (18.5%)	16 (20.3%)	21 (26.6%)	23 (21.9%)	
	Score 2+	32 (7.4%)	6 (6.7%)	4 (5.0%)	9 (11.3%)	4 (4.1%)	9 (8.6%)	
Immunophenotype	Luminal	140 (32.3%)	58 (65.2%)	28 (34.6%)	15 (19.0%)	22 (27.8%)	17 (16.2%)	<0.0001^b^
	Triple-negative	293 (67.7%)	31 (34.8%)	53 (65.4%)	64 (81.0%)	57 (72.2%)	88 (83.8%)	
Ki-67	Mean index (%) ± SD	33.5 ± 17.1	23.4 ± 10.8	33.3 ± 16.3	30.8 ± 16.3	39.0 ± 17.7	40.2 ± 19.5	<0.0001^a^
	Ki-67 <20%	94 (21.7%)	34 (38.2%)	19 (23.4%)	17 (21.5%)	10 (12.6%)	14 (13.3%)	0.0002^b^
	Ki-67 ≥20%	339 (78.3%)	55 (61.8%)	62 (76.6%)	62 (78.5%)	69 (87.4%)	91 (86.7%)	
CK5/6^e^	CK5/6– (<10%)	117 (34.0%)	undetermined	24 (29.6%)	33 (41.8%)	22 (27.8%)	38 (36.2%)	<0.0001^b^
	CK5/6+ (≥10%)	227 (66.0%)	undetermined	57 (70.4%)	46 (58.2%)	57 (72.2%)	67 (63.8%)	
EGFR^e^	EGFR– (<10%)	160 (46.5%)	undetermined	30 (37.0%)	37 (46.8%)	37 (46.8%)	56 (53.3%)	<0.0001^b^
	EGFR+ (≥10%)	184 (53.5%)	undetermined	51 (63.0%)	42 (53.2%)	42 (53.2%)	49 (46.7%)	

a *Analysis of variance*.

b *Chi-square test*.

c *Fisher's exact test*.

d *On a total of 388 cases (45 others from unknown location)*.

e *Only available for stage I–IIIB (invasive) carcinomas (N = 344)*.

*NS, Not Significant; SD, standard deviation*.

The 433 dogs included 96 small-breed dogs (22.2%, weight <10 kg), 105 medium-breed dogs (24.2%, weight 10–25 kg), 87 large-breed dogs (20.1%, weight >25 kg), and 145 (33.5%) dogs of unknown format.

At histology, the mean pathologic tumor size was 16.4 ± 7 mm (median 17 mm, range 2–49 mm, *N* = 309 dogs); in the other 124 cases, the pathologic tumor size could not be precisely determined due to larger size and/or positive margins. At 20 mm threshold, 205 dogs (47.3%) had a tumor larger than 20 mm in diameter (pT2). In 294 dogs (67.9%), the pathologic nodal stage was pNX due to absence of lymph node sampling for histopathology. Nodal stage pN+ (with metastasis of any size) was confirmed in 74 cases (17.1%). The predominant histological types were simple tubulopapillary (*N* = 178; 41.1%), simple solid (*N* = 100; 23.1%), and complex carcinomas (malignant epithelial proliferation associated with benign myoepithelial proliferation, *N* = 93, 21.4%). Among less represented histotypes, there were 5 inflammatory CMCs (1.2%), and 21 anaplastic CMCs (4.8%). The mean mitotic index was 36 ± 29 mitoses in 10 high-power fields (×400, diameter of the field of view 0.55 mm; median 29, range 1–236 mitoses).

Of the studied CMCs, 95 (21.9%) were ER-positive, and 85 (19.6%) were PR-positive. HER2 was scored 0 for 293 CMCs (67.7%), 1+ in 108 (24.9%) cases, and 2+ in the other 32 (7.4%): this cohort did not contain any HER2-positive cases. Thus, 140 CMCs (32.3%) were luminal, and 293 (67.7%) were triple-negative.

### Definition of Histological Stages

**Figure 1:**
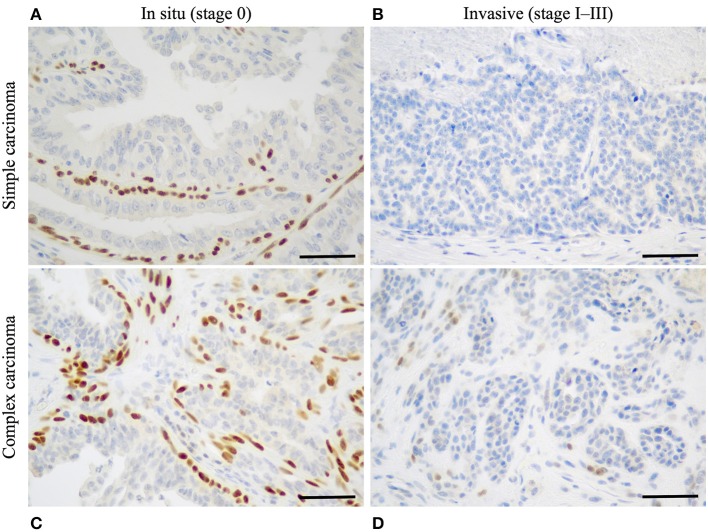
Identification of stage 0 (*in situ*) canine mammary carcinomas by p63 immunohistochemistry. **(A)** Simple mammary carcinoma *in situ*. Presence of a continuous layer of resting or hypertrophic myoepithelial cells surrounding the neoplastic cells and showing strong nuclear p63 immunoreactivity. **(B)** Simple invasive mammary carcinoma. Absence of p63+ myoepithelial cells around the neoplastic cells. **(C)** Complex mammary carcinoma *in situ*. P63 labels the nuclei of spindle-shaped proliferative myoepithelial cells, as well as a continuous layer of hypertrophic myoepithelial cells located in close contact to the neoplastic cells. **(D)** Complex invasive mammary carcinoma. P63 weakly labels spindle-shaped proliferative myoepithelial cells in stromal location, but there is no layer of p63+ myoepithelial cells surrounding the neoplastic cells. P63 immunohistochemistry, original magnification 400×, scale bar = 50 μm.

The first step necessary to define histological stages of CMCs, was to identify CMCs *in situ* (stage 0 CMCs), using p63 IHC. In simple CMCs, i.e., those lacking myoepithelial cell proliferation, p63 stained the nuclei of a continuous layer of myoepithelial cells surrounding neoplastic cells in mammary carcinomas *in situ* (Figure 1A), and was negative in invasive mammary carcinomas (Figure 1B). In complex CMCs, i.e., those with associated myoepithelial cell proliferation, p63 IHC labeled the nuclei of spindle-shaped proliferative myoepithelial cells located at some distance from the neoplastic cells; in complex mammary carcinomas *in situ*, there were also resting or hypertrophic p63+ myoepithelial cells in close vicinity of the neoplastic cells (Figure 1C); in complex invasive mammary carcinomas, there were no p63+ myoepithelial cells adjacent to the carcinoma (Figure 1D).

The second component of the proposed histological staging system was the pathologic tumor size (pT), measured in millimeters on HES-stained sections. By comparison with clinical tumor size (T), pT excluded not only the skin, but also potential areas of mammary gland hyperplasia associated with CMCs; thus, pT was more precise, and smaller, than T. The threshold for pT, >20 mm, is identical to the one used in breast cancer, and was the threshold with best prognostic significance in the present series.

Because a very high proportion of the present CMCs were of unknown pathologic nodal stage (pNX), we have decided to include lymphovascular invasion in the proposed histological staging system, as LVI precedes nodal metastasis, and can be easily assessed at the margins of CMCs. Of note, all of the present cases were analyzed on whole sections (not partial biopsies), thus facilitating LVI identification.

The four components of the proposed histological staging system, invasiveness, pT, LVI, and pN, were all significantly associated with disease-free interval, overall survival, and cancer-specific survival, by univariate analyses (Table 3, upper lines). By multivariate analyses, the four components were independent prognosticators for overall and specific survival, whereas for disease-free interval, pT was not significantly informative (Table 3, lower lines).

**Table 3:** Prognostic value of the parameters included in the histological staging system.

		**Disease-free interval**		**Overall survival**		**Cancer-specific survival**	
		**HR (95% CI)**	***P***	**HR (95% CI)**	***P***	**HR (95% CI)**	***P***
**UNIVARIATE ANALYSES**							
Invasiveness	Invasive vs. *in situ*	5.07 (3.55–7.24)	<0.0001	2.86 (2.28–3.58)	<0.0001	6.78 (4.97–9.26)	<0.0001
Pathologic tumor size	>20 vs. ≤ 20 mm	1.48 (1.06–2.06)	<0.0001	1.82 (1.47–2.26)	<0.0001	1.81 (1.35–2.42)	<0.0001
Lymphovascular invasion	LVI+ vs. LVI–	3.87 (2.66–5.63)	<0.0001	3.01 (2.34–3.87)	<0.0001	4.48 (3.22–6.22)	<0.0001
Pathologic nodal stage	pN+ vs. pN0–PNX	2.94 (1.72–5.01)	<0.0001	2.51 (1.75–3.60)	<0.0001	3.19 (2.01–5.04)	<0.0001
**MULTIVARIATE ANALYSES**							
Invasiveness	Invasive vs. *in situ*	2.93 (1.53–5.63)	0.0013	1.80 (1.28–2.51)	0.0006	3.53 (1.86–6.71)	0.0001
Pathologic tumor size	>20 vs. ≤ 20 mm	1.22 (0.88–1.69)	0.2281	1.59 (1.28–1.97)	<0.0001	1.45 (1.09–1.94)	0.0102
Lymphovascular invasion	LVI+ vs. LVI–	2.67 (1.85–3.85)	<0.0001	2.25 (1.76–2.87)	<0.0001	2.95 (2.14–4.07)	<0.0001
Pathologic nodal stage	pN+ vs. pN0–PNX	1.58 (1.05–2.36)	0.0260	1.56 (1.17–2.08)	0.0020	1.66 (1.18–2.34)	0.0034

### Differences in Initial Presentation According to Histological Stages

At initial presentation, female dogs with mammary carcinoma *in situ* (Stage 0, *N* = 89) were significantly younger at diagnosis (mean 9.7 ± 2.2 years, median 10.2 years, range 4.5–14.4 years) than female dogs with invasive CMCs (mean 11.0 ± 2.1 years, median 11.0 years, range 3.6–16.3 years, *p* < 0.001, *N* = 344). Stage 0 CMCs were of smaller pathologic tumor size (mean 12.8 ± 6.3 mm, median 12.0 mm, range 2–30 mm) than invasive CMCs (mean 17.8 ± 6.9 mm, median 18.0 mm, range 2–49 mm, *p* < 0.001). Stage 0 CMCs were also of lower histological grade (66% grade I, 29% grade II, 5% grade III) than invasive CMCs (6% grade I, 30% grade II, 64% grade III, *p* < 0.0001). There was higher ER expression in CMCs *in situ* (of which 44% were ER+) than in invasive CMCs (of which 16% were ER+, *p* < 0.0001), and also higher PR expression (*P* < 0.0001): 51% out of the CMCs *in situ* (45/89) were PR+, compared to 12% out of the invasive CMCs (40/344). The mean ER index was 11.7 ± 10.8% in stage 0 CMCs compared to 6.1 ± 13.4% in invasive CMCs (*p* < 0.001). The mean PR index was 14.0 ± 12.7% in stage 0 CMCs compared to 5.5 ± 14.9% in invasive CMCs (*p* < 0.001). Finally, CMCs *in situ* showed a lower Ki-67 proliferation index (mean 23 ± 11%, median 23%) than invasive CMCs (mean 36 ± 17%, median 35%, *p* < 0.001).

Among invasive CMCs, there were also significant differences in initial presentation according to histological stages (Table 2). Stage I–II CMCs (*N* = 160) were diagnosed at younger age (mean 10.7 ± 2.1 years, median 10.8, range 3.6–16.1 years) than stage IIIA–IIIB CMCs (mean 11.2 ± 2.1 years, median 11.1, range 6.2–16.3 years, *p* = 0.046, *N* = 184). Stage IIIA–IIIB CMCs were more commonly diagnosed with positive margins (111/184, 60%) than stage I–II CMCs (44/160, 28%, *p* < 0.0001). Stage IIIA–IIIB CMCs were of higher histological grade (1% grade I, 28% grade II, 71% grade III) than stage I–II CMCs (11% grade I, 32% grade II, 57% grade III, *p* = 0.0002). Compared to stage I–II CMCs, stage IIIA-IIIB CMCs were more commonly associated with moderate to severe peritumoral inflammation (59% of stage IIIA-IIIB CMCs with inflammation vs. 35% of stage I–II CMCs, *p* < 0.0001). The PR index was significantly lower in stage IIIA–IIIB CMCs (mean 3.7 ± 11.6%, median 0%) than in stage I–II CMCs (mean 7.6 ± 18.8%, median 0%, *p* = 0.015). Stage IIIA–IIIB CMCs had a higher Ki-67 proliferation index (mean 40 ± 19%, median 38%) than stage I–II CMCs (mean 32 ± 15%, median 32%, *p* < 0.001). However, there were no significant differences in pathologic tumor size between stage I–II CMCs (mean 17 ± 6 mm) and stage IIIA–IIIB CMCs (mean 18 ± 8 mm, *p* = 0.223), and no significant differences in CK5/6 and EGFR expression between stage I–II CMCs and stage IIIA–IIIB CMCs.

### Disease-Free Interval by Histological Stage

The median DFI was 1,720 days (4 years and 8.5 months). Cancer progression (locoregional recurrence and/or distant metastasis) was recorded in 27% of dogs at 1-year post-diagnosis, and 36% at 2 years.

**Figure 2:**
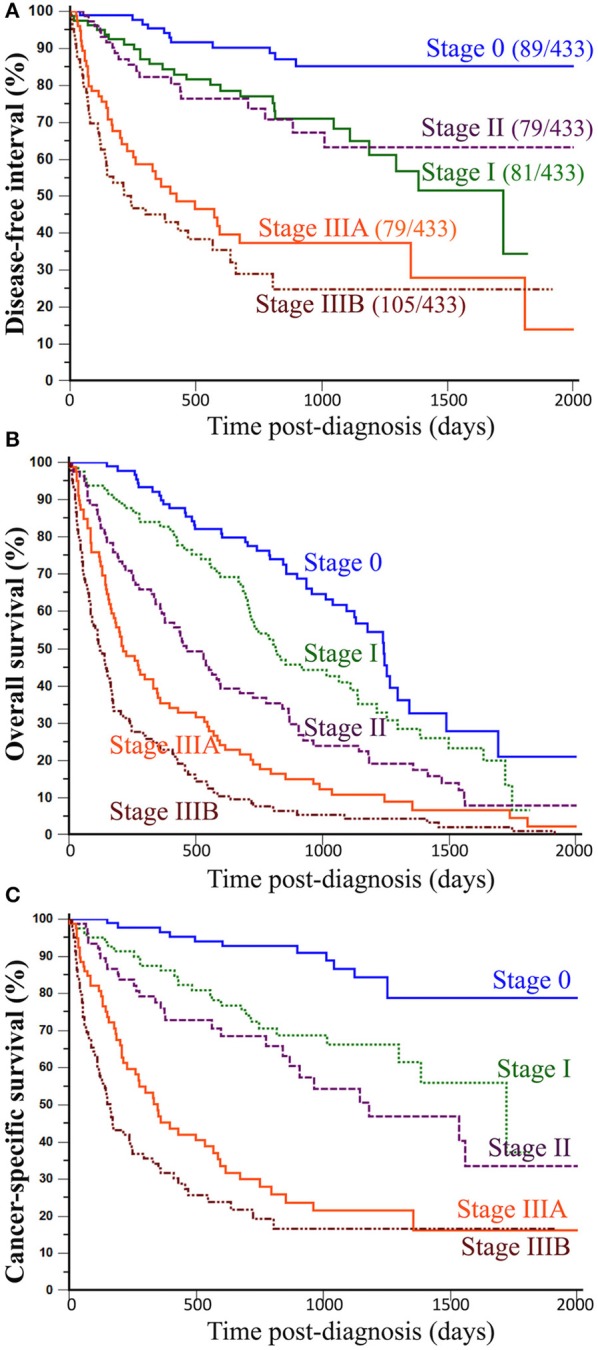
Association between histological stages of CMCs and outcomes of canine patients. **(A)** Disease-free interval. The probability of locoregional recurrence and/or distant metastasis significantly increased from stage 0 to stage IIIB CMCs, however without significant differences between stage I and stage II CMCs. **(B)** Overall survival. All-cause mortality of female dogs with mammary carcinoma significantly increased with increasing histological stage at presentation. **(C)** Cancer-specific survival. The probability of dying from cancer significantly increased with histological stage. Kaplan-Meier curves. See Table 4 for corresponding hazard ratios and *p*-values.

When split by histological stage, median disease-free intervals were not reached for stage 0 and stage II CMCs, were 1,720 days for stage I CMCs, 423 days for stage IIIA, and 241 days for stage IIIB CMCs. The probabilities of cancer progression within 1 year post-diagnosis were 5% for stage 0, 15% for stage I, 18% for stage II, 47% for stage IIIA, and 55% for stage IIIB CMCs. Compared to stage 0 CMCs (HR = 1.00, reference), the probabilities of cancer progression were 2.91 times higher for stage I CMCs (*p* = 0.0029), 2.68 times higher for stage II CMCs (*p* = 0.0094), 8.07 times higher for stage IIIA CMCs (*p* < 0.0001), and 11.46 times higher for stage IIIB CMCs (*p* < 0.0001; Table 4 and Figure 2A).

**Table 4:** Prognostic factors of canine mammary carcinomas.

**Univariate analyses**		**Disease-free interval**		**Overall survival**		**Cancer-specific survival**	
		**HR (95% CI)**	***P***	**HR (95% CI)**	***P***	**HR (95% CI)**	***P***
Multicentricity	Single vs. multiple	0.49 (0.29–0.83)	0.0006	–	NS	–	NS
Margin status	Positive vs. negative	1.66 (1.18–2.34)	0.0017	1.91 (1.53–2.39)	<0.0001	1.93 (1.43–2.61)	<0.0001
Histological type	Inflammatory vs. others	6.81 (1.63–28.54)	0.0090	11.56 (4.66–28.67)	<0.0001	14.35 (5.71–36.07)	<0.0001
	Anaplastic vs. others	2.11 (1.01–4.38)	0.0468	3.45 (2.18–5.46)	<0.0001	3.29 (1.87–5.78)	<0.0001
Tumor-associated inflammation	Moderate to severe vs. absent to mild	1.77 (1.26–2.49)	0.0004	1.65 (1.33–2.06)	<0.0001	2.06 (1.53–2.78)	<0.0001
Dermal invasion	Yes vs. no	–	NS	1.46 (1.14–1.86)	0.0007	1.69 (1.22–2.34)	0.0004
Cutaneous ulceration	Yes vs. no	–	NS	1.66 (1.14–2.42)	0.0009	1.91 (1.17–3.13)	0.0007
Histological grade	III vs. I	4.19 (2.34–7.51)	<0.0001	3.63 (2.57–5.12)	<0.0001	5.30 (3.10–9.05)	<0.0001
	II vs. I	2.85 (1.54–5.26)	0.0009	2.25 (1.56–3.25)	<0.0001	2.68 (1.51–4.76)	0.0008
ER	ER+ vs. ER–	0.49 (0.34–0.71)	0.0015	0.59 (0.47–0.75)	0.0001	0.46 (0.33–0.63)	0.0001
PR	PR+ vs. PR–			0.71 (0.55–0.91)	0.0159	0.58 (0.41–0.82)	0.0015
Ki-67	≥20% vs. <20%	2.19 (1.51–3.17)	0.0008	1.85 (1.49–2.33)	<0.0001	2.50 (1.85–3.33)	<0.0001
Triple-negative vs. luminal		1.74 (1.19–2.52)	0.0109	1.37 (1.07–1.76)	0.0186	2.07 (1.30–3.29)	<0.0001
Histological stage	IIIB vs. 0	11.46 (5.94–22.12)	<0.0001	6.66 (4.69–9.48)	<0.0001	16.58 (8.75–31.44)	<0.0001
	IIIA vs. 0	8.07 (4.13–15.74)	<0.0001	3.95 (2.74–5.71)	<0.0001	10.59 (5.52–20.30)	<0.0001
	II vs. 0	2.68 (1.27–5.62)	0.0094	2.40 (1.65–3.47)	<0.0001	4.39 (2.20–8.74)	<0.0001
	I vs. 0	2.91 (1.44–5.88)	0.0029	1.56 (1.06–2.29)	0.0238	3.05 (1.51–6.13)	0.0018

*NS, not significant*.

By univariate analysis, eight parameters other than histological stage were significantly associated with disease-free interval (Table 4), i.e., multicentricity, margin status, an inflammatory or anaplastic histological type, tumor-associated inflammation, the histological grade, ER expression, the Ki-67 proliferation index, and the immunophenotype (luminal vs. triple-negative).

In the 344 female dogs with invasive mammary carcinoma, the histological stage was a significant prognostic factor associated with disease-free interval by multivariate analysis (HR = 3.60 for stage IIIB, HR = 2.66 for stage IIIA CMCs compared to stage I; Table 5), with 2 independent covariates (*p* < 0.0001): the immunophenotype (HR = 1.64 for triple-negative CMCs compared to luminal), and the Ki-67 proliferation index (HR = 1.70 for CMCs with Ki-67 index ≥20% compared to <20%).

**Table 5:** Prognostic value of the histological staging system applied to invasive CMCs.

**Multivariate analyses**		**Disease-free interval**		**Overall survival**		**Cancer-specific survival**	
		**HR (95% CI)**	***P***	**HR (95% CI)**	***P***	**HR (95% CI)**	***P***
Histological stage	IIIB vs. I	3.60 (2.22–5.85)	<0.0001	3.59 (2.55–5.04)	<0.0001	4.47 (2.81–7.11)	<0.0001
	IIIA vs. I	2.66 (1.61–4.37)	0.0001	2.27 (1.59–3.23)	<0.0001	2.98 (1.85–4.79)	<0.0001
	II vs. I	0.84 (0.46–1.52)	0.5686	1.46 (1.02–2.09)	0.0376	1.28 (0.75–2.16)	0.3536
Multicentricity	Single vs. multiple	–	NS	0.60 (0.44–0.82)	0.0017	–	
Tumor-associated inflammation	Moderate to severe vs. absent to mild	–	NS	1.27 (1.01–1.60)	0.0409	1.42 (1.05–1.90)	0.0228
Histological grade	III vs. I–II	–	NS	1.30 (1.01–1.66)	0.0369	1.39 (1.00–1.92)	0.0469
Ki-67	≥20% vs. <20%	1.70 (1.00–2.88)	0.0470	–	NS	–	NS
	Triple-negative vs. luminal	1.64 (1.05–2.56)	0.0273	–	NS	–	NS

*NS, not significant*.

### Overall Survival by Histological Stage

During the follow-up period, 352 dogs (81.3%) died. The median overall survival time was 496 days (1 year and 4.3 months; range, 2–2,663 days). The mortality rate was 43% at 1 year and 62% at 2 years post-diagnosis. Death was unrelated to cancer in 73 dogs (20.7%), from unknown causes in 87 dogs (24.7%), and attributable to the mammary carcinoma in 192 dogs (54.5%).

The proposed histological stages were significantly associated with all-cause mortality of female dogs of the present study. Indeed, median overall survival times were 1,241 days for stage 0 CMCs (3 years and 4.8 months), 813 days for stage I CMCs (2 years and 2.7 months), 464 days for stage II CMCs (1 year and 3.2 months), 213 days for stage IIIA CMCs (7.0 months), and only 123 days for stage IIIB CMCs (4.0 months). The probabilities of death from all causes within 1 year post-diagnosis were 10% for stage 0 CMCs, 17% for stage I CMCs, 39% for stage II CMCs, 65% for stage IIIA CMCs, and 77% for stage IIIB CMCs. Compared to stage 0 CMCs (HR = 1.00, reference), the probabilities of dying from any cause were 1.56 times higher for stage I CMCs (*p* = 0.0238), 2.40 times higher for stage II CMCs (*p* < 0.0001), 3.95 times higher for stage IIIA CMCs (*p* < 0.0001), and 6.66 times higher for stage IIIB CMCs (*p* < 0.0001; Table 4 and Figure 2B).

Apart from histological stage, 10 other parameters were significantly associated with overall survival (Table 4), i.e., margin status, an inflammatory or anaplastic histological type, tumor-associated inflammation, dermal invasion, cutaneous ulceration, the histological grade, ER and PR expression, the Ki-67 proliferation index, and the immunophenotype.

In the 344 patients with invasive CMCs, the histological stage (HR = 3.59 for stage IIIB, HR = 2.27 for stage IIIA, HR = 1.46 for stage II CMCs compared to stage I) was a significant predictor of overall survival by multivariate analysis (Table 5), with 3 independent covariates (*p* < 0.0001): the histological grade (HR = 1.30 for grade III CMCs compared to grade I–II), tumor-associated inflammation (HR = 1.27 when moderate to severe compared to absent to mild), and multicentricity (HR = 0.60 for single CMCs compared to multicentric CMCs).

### Specific Survival by Histological Stage

The median time to death attributable to cancer was 1,124 days (3 years and 1.0 month; range, 2–1,720 days). The cancer-related death rate was 33% at 1 year and 43% at 2 years post-diagnosis. However, these survival probabilities were highly dependent on histological stage at diagnosis: median specific survival times were not reached for stage 0 CMCs, 1,720 days for stage I CMCs (4 years and 8.5 months), 1,181 days for stage II CMCs (3 years and 2.8 months), 348 days for stage IIIA CMCs (11.4 months), and 163 days for stage IIIB CMCs (5.3 months). The probabilities of cancer-related death within 1-year post-diagnosis were 3% for stage 0 CMCs, 14% for stage I CMCs, 24% for stage II CMCs, 55% for stage IIIA CMCs, and 68% for stage IIIB CMCs. Compared to stage 0 CMCs (HR = 1.00, reference), the probabilities of dying from cancer were 3.05 times higher for stage I CMCs (*p* = 0.0018), 4.39 times higher for stage II CMCs (*p* < 0.0001), 10.59 times higher for stage IIIA CMCs (*p* < 0.0001), and 16.58 times higher for stage IIIB CMCs (*p* < 0.0001; Table 4 and Figure 2C).

By univariate analysis, 10 parameters other than histological stage were significantly associated with cancer-specific survival (Table 4): margin status, an inflammatory or anaplastic histological type, tumor-associated inflammation, dermal invasion, cutaneous ulceration, the histological grade, ER and PR expression, the Ki-67 proliferation index, and the immunophenotype (luminal vs. triple-negative).

In the 344 female dogs with invasive mammary carcinomas, the risk of cancer-related death was predicted by 3 independent prognostic factors by multivariate analysis (*p* < 0.0001; Table 5): the histological stage (HR = 4.47 for stage IIIB, HR = 2.98 for Stage IIIA compared to stage I), the histological grade (HR = 1.39 for grade III CMCs compared to grade I–II), and tumor-associated inflammation (HR = 1.42 when moderate to severe compared to absent to mild). These results indicated that the proposed histological system, the histological grade, and tumor-associated inflammation were the strongest prognostic factors associated with cancer-related death probabilities in dogs with invasive mammary carcinomas.

## Discussion

The prognostic value of a new histological staging system was evaluated in this study, in the largest cohort of female dogs with mammary carcinoma described so far (*N* = 433). There have been previous reports of a staging system for CMCs relying on local invasiveness, lymphovascular invasion, lymph node metastasis, and distant metastasis, which was significantly associated with recurrence-free interval and overall survival in 134 female dogs, but this system was improperly designated “grade,” and only 32 bitches had malignant tumors (the other 102 had benign mammary tumors) (35). In the present study, this is the first time that a complete histological staging system is proposed that takes into account mammary carcinomas *in situ* (stage 0) and the unknown pathologic nodal stage category pNX, and provides information regarding disease-free interval, overall survival, and cancer-specific survival according to histological stage. Parameters of this histological staging system were largely inspired by those used in breast cancer staging (18) but were drastically simplified in order to facilitate its routine application in veterinary medicine.

Historically, in order to stage canine mammary tumors, i.e., to describe cancer spread within the host, the clinical system TNM proposed by the World Health Organization in 1980 (7) and modified thereafter (6), was used to specify the prognosis of dogs. Multiple studies have proven its ease of use and its effectiveness in predicting patient outcomes (5, 8–11), although the TNM system is perfectible. Regarding clinical tumor size, the cut-offs of 3 cm (10) and 5 cm (5, 8) have been significantly associated with overall survival, and Peña et al. reported that the thresholds of <1 and ≥3 cm were also significantly associated with overall survival in a prospective study of 65 female dogs with CMC (9). The main drawback of the clinical tumor size measured by caliper is that it may comprise skin and adipose tissue thickness, as well as eventual mammary hyperplasia adjacent to the carcinoma. This is the reason why we referred here to the pathologic tumor size (pT) instead of clinical tumor size, as recommended for breast cancer. The threshold of >20 mm, identical to the one used in breast cancer staging (18), had significant prognostic value in the present cohort, in terms of disease-free interval (HR = 1.48, 95%CI 1.06–2.06; *P* < 0.0001), overall survival (HR = 1.82, 95%CI 1.47–2.26; *P* < 0.0001), and cancer-specific survival (HR = 1.81, 95%CI 1.35–2.42; *P* < 0.0001). The 2-years overall survival rates were 53% for pT1 CMCs (≤20 mm) and 43% for pT2 CMCs (>20 mm), in agreement with survival differences observed in women with breast cancer: reported 5-years survival rates are 100% for carcinomas ≤ 20 mm in diameter, and 93% for larger carcinomas^1^. In breast cancer staging, other pT categories are defined, including pT3 >50 mm in greatest dimension, and pT4 of any size, with direct extension to the chest wall and/or to the skin (ulceration or skin nodules) (18). As we have found that cutaneous ulceration was a significant prognostic factor in terms of overall survival (HR = 1.66, 95%CI 1.14–2.42; *P* = 0.0009) and cancer-specific survival (HR = 1.91, 95%CI 1.17–3.13; *P* = 0.0007, univariate analyses), in agreement with previous reports in CMCs (11), it is likely that a pT4 category can be defined for CMCs. In breast cancer, pT4 tumors are stage IIIB (pT4, N0 to N2, M0), stage IIIC (any pT, N3, M0), or stage IV (any pT, any N, M1) (18). For the sake of simplicity, we did not use a pT4 category in the present histological system, as this would have led to a 7-stage system (instead of 5 stages currently), with addition of a stage IIB (pT4, LVI–, pN0–pNX) and a stage IIIC (pT4, LVI+, and/or pN+).

One of the major changes introduced in the present histological staging system is the recognition of a specific category for mammary carcinomas *in situ* (stage 0), as exists in breast cancer: stage 0 breast cancers are pTis (carcinoma *in situ*), N0, M0 (18). Local invasiveness is easier to detect using immunohistochemical staining of myoepithelial cells rather than on HES-stained slides, and multiple markers have been proposed for canine myoepithelial cells, including alpha smooth muscle actin, calponin, CD10, keratins 5/6 and 14, and p63 (13–15, 36–38). Double immunostaining to p63 and calponin is the method of choice for myoepithelial cell identification in CMCs (14, 38), whereas p63 alone is routinely used in breast cancer pathology (28). Using the same antibody to p63 as the one in the present study (clone 4A4), Łopuszynski et al. were able to differentiate canine mammary carcinomas *in situ* from simple or complex invasive CMCs with good sensitivity, and concluded that p63 is a sensitive and more specific marker of myoepithelial cells in canine mammary tumors compared with calponin (13). The distinction between CMCs *in situ* and invasive CMCs seems to us of paramount importance. First, they significantly differed by initial presentation, as CMCs *in situ* were diagnosed at younger age, were smaller, of lower histological grade, had higher ER and PR expression, and a lower proliferation index, than invasive CMCs. Most importantly, CMCs *in situ* were significantly associated with much better outcomes than invasive CMCs, in terms of cancer progression (disease-free interval), all-cause mortality (overall survival), and death attributable to cancer (specific survival). The 2-years overall survival rates were 93% for dogs with CMC *in situ* compared to 47% for dogs with invasive CMC (*P* < 0.0001), in agreement with reported differences in 5-years overall survival rates in breast cancer: 100% for patients with carcinoma *in situ* vs. 89.7% for those with invasive carcinomas^1^. Lastly, CMCs *in situ* may not be indications for adjuvant chemotherapy, contrary to invasive CMCs with nodal and/or distant metastasis.

The major weakness of the TNM staging system for CMCs is clinical evaluation of nodal stage (N) by palpation, cytology and/or medical imaging, because of low sensitivity. In clinically node-negative breast cancer, the sensitivity of preoperative ultrasound for nodal metastasis detection is only 7.4%, and the false-positive rate is 80%, thus sentinel lymph node biopsy remains the gold standard for nodal staging in early breast cancer (39). The pathologic nodal status (pN) used in the present staging system was assisted by anti-pancytokeratin AE1/AE3 immunohistochemical labeling of epithelial cells, a method that increases the sensitivity of small metastasis detection, as recommended for CMCs (14, 21, 29). Indeed, these antibodies allow detecting occult micrometastases in 2.8–9.2% of lymph nodes that are free of metastasis by conventional histological evaluation (20, 29). The main advantage of this immunochemical method is to facilitate isolated tumor cell detection (<0.2 mm in diameter or 200 cells): isolated tumor cell detection increased by 35.2% when compared with histological examination according to Coleto et al. (20). A recent prospective study of 936 pT1N0M0 breast cancer patients confirmed that isolated tumor cells in the sentinel lymph node were associated with unfavorable survival, justifying their detection by cytokeratin immunohistochemistry (40). In the present study, we defined positive nodal stage (pN+) by the presence of isolated tumor cells, micrometastases or macrometastases, whereas the pathologic nodal stage of breast cancer is still pN0 when only isolated tumor cells are present within the sentinel lymph node (18). The reason is that the draining lymph node examined in the present study was probably not the sentinel lymph node, as sentinel lymph node mapping is not routinely performed in veterinary oncology. Thus, isolated tumor cells in a higher-level lymph node may correspond to the presence of a micro- or macrometastasis in the sentinel lymph node, i.e., a positive nodal stage. According to Coleto et al. however, the presence of isolated tumor cells was not significantly associated with a shorter overall survival in dogs with CMC, and isolated tumor cells may not be included in the definition of positive nodal stage (20). Szczubiał et al. have shown that pN is significantly associated with disease-free survival and overall survival of dogs with CMC, with a poorer outcome associated with macrometastases vs. micrometastases vs. absence of metastases (respective 2-years overall survival rates of 7.1, 50, and 51.7%) (21). In human breast cancer, a mean of 18 axillary lymph nodes may be assessed in case of axillary lymph node dissection (41), and the pathologic nodal stage is one of the strongest prognostic factors for breast cancer, with reported 5-years overall survival rates of 98.6% for localized tumors and 84.4% for breast cancers with nodal metastases^1^.

In veterinary medicine, the draining lymph node is not always surgically removed with CMCs; for instance in this series, 294 dogs (67.9%) had a pNX pathologic nodal stage (unsampled lymph node). This is the reason why lymphovascular invasion was added to nodal status in the present study, as a reflection of metastatic spread via the blood or the lymphatics. Actually, in our study, LVI appeared to be a sensitive (85.6%) and specific (73.0%) indicator of lymph node metastasis. In human breast cancers, LVI has 30.8% sensitivity, and 90.9% specificity to predict the presence of isolated tumor cells in the sentinel lymph node (42). We confirmed here that LVI associated with CMCs had a strong negative prognostic value, as previously reported in dogs in terms of overall survival by univariate analyses (5, 23, 24) and by multivariate analysis, independently of the histological grade (24), or independently of clinical stage, cutaneous ulceration, and surgical margins (11).

We then checked that the four prognostic parameters described above, i.e., invasiveness, pathologic tumor size, lymphovascular invasion, and pathologic nodal stage, had independent prognostic value in dogs of the present series, which was true for overall and cancer-specific survival. For disease-free interval, the pathologic tumor size was not significantly informative compared to invasiveness, lymphovascular invasion, and the nodal status. We have then designed a 5-stage system with a significant prognostic value. This system is largely inspired by breast cancer staging system, which also has strong prognostic value: the reported 5-years survival rates are 99.1% for stage I breast cancers, 98.0% for stage IIA (*P* = 0.002), 95.6% for stage IIB, 95.4% for stage IIIA, and 79.5% for stage IIIC breast cancers (*P* < 0.0001) (43). By comparison, the 2-years overall survival probabilities were much lower in female dogs of the present series, as none of them received adjuvant therapy: 78% for stage 0, 57% for stage I, 37% for stage II, 19% for stage IIIA, and 8% for stage IIIB CMCs. Of note, the histological stages used for breast cancer and those proposed for dogs with CMCs are not identical, as breast cancer staging uses 4 pT categories and 4 pN categories, whereas we have opted for a much simpler system for CMCs, in order to facilitate its routine application.

The present histological staging system for dogs with CMCs has some limitations. First, it was designed for stage I–III CMCs, i.e., those without distant metastasis at diagnosis. In breast cancer staging, stage IV corresponds to any pT, any pN, M1, as also defined for CMCs in the WHO staging system (7). Secondly, the present histological staging system only applies to mammary carcinomas, not carcinosarcomas, not sarcomas, as the current staging system for canine mammary malignant tumors (6). Thirdly, we referred to the seventh edition of breast cancer staging edited by the American Joint Committee on Cancer (18), but there has been an update with an eighth edition published in 2017. This update does not significantly modify the pT and pN categories, and the anatomical stages of breast cancer, but takes into account pT, pN, M, ER, PR, HER2, and the histological grade, combined in a “pathologic prognostic stage” for patients who received surgery as initial treatment (43, 44). There is therefore room for further improvements of the present histological staging system for CMCs, to better assess patient outcomes, however with supplementary costs induced by immunohistochemical evaluation of ER, PR, and HER2 expression.

In conclusion, the proposed system for histological staging of CMCs (invasiveness, pT, LVI, pN) was inspired from human oncology with drastic simplification, in order to be routinely applicable, and to significantly refine prognosis assessment. We hope that this system could be used in the near future for patient randomization in clinical trials evaluating adjuvant chemotherapy for CMCs.

## Data Availability Statement

The datasets generated for this study are available on request to the corresponding author.

## Ethics Statement

The animal study was reviewed and approved by CERVO, Comité d'Ethique en Recherche clinique et épidémiologique Vétérinaire d'Oniris, Oniris, Nantes Atlantic College of Veterinary Medicine, Food Science and Engineering, Nantes, France. Written informed consent was obtained from the owners for the participation of their animals in this study.

## Author Contributions

JA and FN: conceptualization, project administration, supervision. FC, DL, and FN: format analysis, investigation, methodology. JA and FN: funding acquisition. FC and FN: writing, original draft preparation. FC, JA, DL, and FN: writing, review and editing.

### Conflict of Interest

The authors declare that the research was conducted in the absence of any commercial or financial relationships that could be construed as a potential conflict of interest.

## Footnotes

^1^SEER Cancer Statistics Review 1975–2010. https://seer.cancer.gov/archive/csr/1975_2010/results_merged/sect_04_breast.pdf
